# Homogenization Equivalence Modeling of Honeycomb Bending Considering Regional Deformation Differences

**DOI:** 10.3390/polym18161970

**Published:** 2026-08-13

**Authors:** Wangzi Liu, Guangjie Huang, Xianmo Wang, Yingwei Yu, Zhihui Liu, Haixin Guan, Jingping Zhu, Yu Wang

**Affiliations:** 1 College of Materials Science and Engineering, Chongqing University, Chongqing 400044, China; 2 Changhe Aircraft Industry (Group) Co., Ltd., Jingdezhen 333000, China; 3 Jiang Xi Aircraft Airworthiness Certification Center, CAAC, Nanchang 330098, China; 4 College of Electromechanical Engineering, Suqian University, Suqian 223800, China

**Keywords:** aramid paper honeycomb, homogenization equivalence, cell deformation, finite element simulation

## Abstract

A Nomex aramid paper honeycomb sandwich structure is the core material of the main load-bearing structures in aviation. Macroscopic full-scale refined modeling faces the problems of large mesh quantity and high calculation cost. Homogenization equivalence is the core path to achieve its efficient simulation design. Most of the existing mature equivalent models are based on the ideal assumption that the honeycomb always remains macroscopically straight, without considering the equivalent performance changes caused by the morphological distortion of micro-cells under bending conditions. Therefore, it is difficult to support a high-precision simulation of large-curvature special-shaped honeycomb sandwich structures. This paper takes the over-stretched rectangular lattice aramid honeycomb as the research object. The mechanical parameters of the matrix are calibrated through experiments, and the reliability of the fine shell finite element model is verified (the maximum error of the end-face strain characteristics between simulation and the DIC test is less than 10%). A customized finite element sample matrix for compression bending is designed, and the angle distribution laws of honeycomb cells under different thicknesses and different bending curvatures are extracted. It is found that the cell angle shows a linear change trend along the wall-thickness direction, which is only strongly correlated with the initial geometric parameters and the bending radius. Finally, a semi-empirical model that can quickly predict the morphology of bent honeycomb cells is obtained through fitting. Verified by the glass compression-molding visualization experiment, the maximum relative error of the predicted cell angle is only 5.05%. This research establishes a rapid characterization method for the deformation of honeycomb cells under bending deformation, providing theoretical support for the microscopic homogenization equivalent modeling of curved honeycomb sandwich structures.

## 1. Introduction

Honeycomb sandwich structures have become the preferred solution for core load-bearing components in fields such as aerospace and rail transit. Among them, Nomex aramid honeycomb cores are the mainstream core materials for the main load-bearing sandwich structures of aircraft. Honeycomb is a high-frequency periodic structure. If all the microscopic cells of the full-scale honeycomb structure are directly modeled, this will generate a huge number of meshes and bring extremely high computational costs, which cannot meet the requirements of engineering design efficiency. Therefore, the homogenization equivalent method is the core technology for transforming periodic microporous cores into homogeneous continuous media, deriving macroscopic equivalent parameters, and achieving efficient simulation and design of honeycomb sandwich structures. It has long received considerable attention from the academic and industrial circles.

Three core technology paths have been formed in the research of this field so far. The early classic analytical framework laid the foundation for the derivation of equivalent parameters of regular hexagonal honeycombs. However, due to the simplification of mesoscopic features such as node deformation and wall-thickness distribution, there are obvious limitations in the prediction accuracy of actual engineering honeycombs [1,2]. Then, the improved analytical methods have gradually incorporated engineering features such as node stiffness, uneven wall thickness, non-uniformity of resin impregnation, and manufacturing defects, significantly improving the prediction accuracy of conventional engineering honeycombs [3,4,5]. Numerical and multi-scale homogenization methods solve the problems of high-efficiency and high-precision equivalence of complex non-uniform honeycombs, honeycombs with random manufacturing defects, and gradient/new functional honeycombs through means such as FFT acceleration, multi-scale coupling, and random analysis [6,7,8,9]. In recent years, the emerging data-driven methods have further relied on machine learning to achieve real-time prediction of honeycomb equivalent performance, expanding the application scenarios of the homogenization method [10,11,12].

However, at present, the vast majority of mature honeycomb equivalent models are established based on the ideal assumption that the honeycomb always maintains macroscopic flatness, without considering the morphological changes in the cells when the honeycomb undergoes macroscopic bending. As the core material of sandwich structures, in actual service scenarios such as aviation and rail transit, in order to meet the increasingly high requirements for aerodynamic characteristics, more and more curved honeycomb sandwich structures have emerged. In fact, during the macroscopic bending process of the honeycomb, the geometric configuration of the microscopic cells will inevitably be distorted [13,14,15,16]. Even if the deformation at the cell scale is within a limited range, it will cause varying degrees of changes in the core properties of the honeycomb, such as equivalent mechanics and thermodynamics [17,18,19,20]. Therefore, the equivalent parameter system of traditional flat honeycombs cannot be directly applied to the homogenization equivalence of curved honeycombs. The focus of the current research in the academic community is mainly on the correction of mesoscopic features of flat honeycombs and the numerical accelerated equivalence of non-homogeneous honeycombs. A mature research framework for the homogenization modeling method of honeycombs after bending deformation has not yet been formed, which makes it difficult to support high-precision simulation of honeycomb sandwich structures with bending deformation and meet the design requirements of high-reliability scenarios such as the main load-bearing sandwich structures in aviation. The research on the homogenization equivalence theory of curved honeycombs has clear theoretical complement value and engineering application significance.

**Figure 1:**
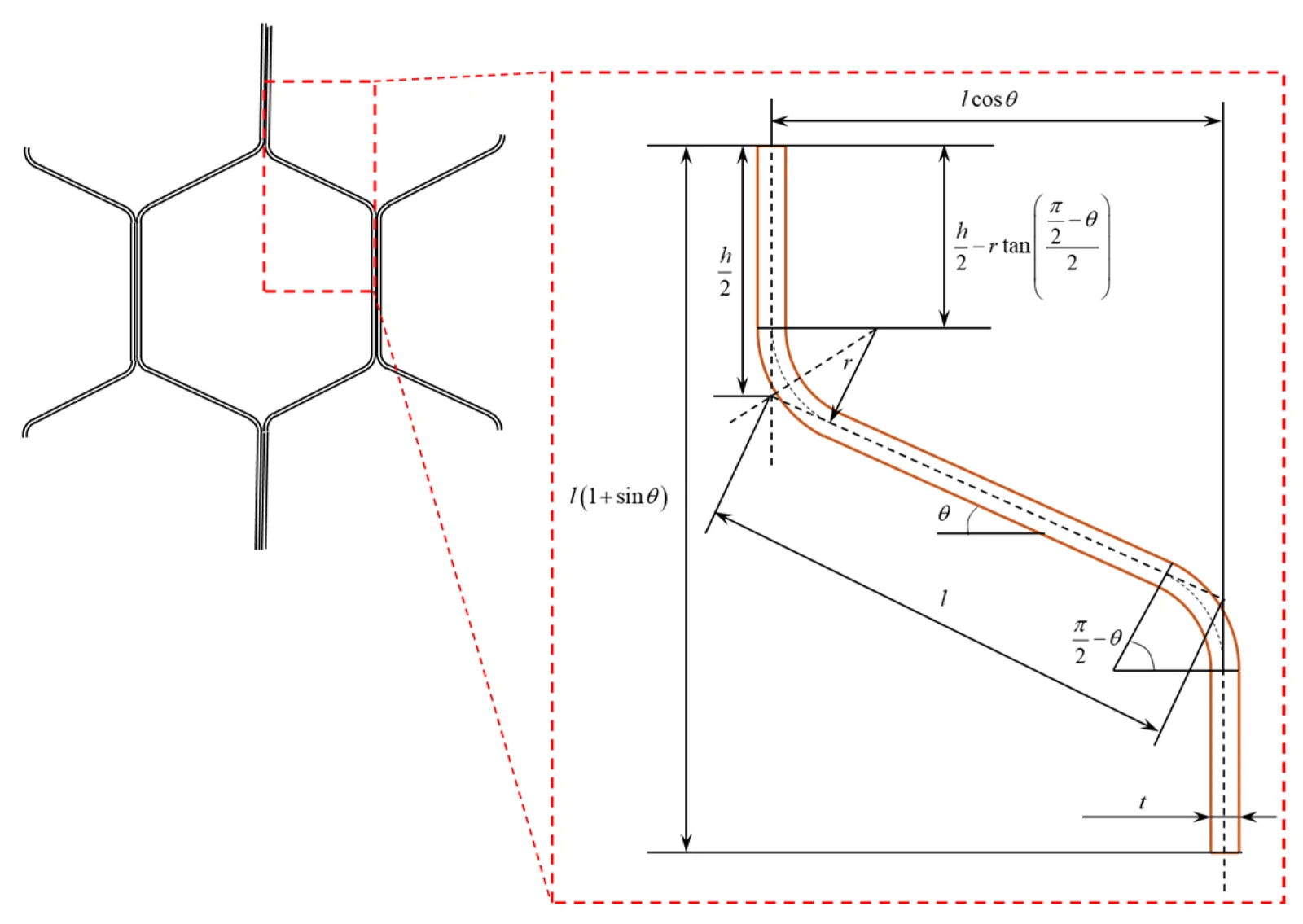
Honeycomb cell structure.

Therefore, it is worth investigating which homogenization and equivalence method should be adopted when a honeycomb undergoes bending deformation. This paper will draw upon Harkati’s homogenization equivalence model for honeycombs to conduct relevant research, as shown in Figure 1 [17]. The research in this paper aims to establish an accurate scaled finite element model of honeycombs through the combination of experiments and simulations. Then, a series of investigations on the cell deformation response of bent honeycombs are carried out using the scaled honeycomb finite element model. Finally, by fitting the series of simulation results with functions, a semi-empirical prediction model for the cell deformation response of honeycombs based on the initial shape of the honeycombs and the bending radius of the honeycombs is summarized.

## 2. Experiment

### 2.1. Materials

Experimental honeycomb was sourced from AVIC Composite Materials Co., Ltd., Jingdezhen, China. The matrix material of honeycomb core is aramid paper that is fully cured after being impregnated with epoxy resin.

Research indicates that the matrix material of honeycomb cores can be considered an isotropic material. The mechanical properties of the honeycomb core matrix material were determined using uniaxial tensile testing. The specimens were prepared in accordance with the TAPPI T494 test standard, with a thickness of 0.14 mm. The mechanical properties of the matrix material, as determined by testing, are shown in Table 1.

The specifications and parameters of the aramid paper honeycomb used in this study are shown in Table 2.

This is a honeycomb structure with rectangular cells. During the manufacturing process, the original hexagonal cells are transformed into rectangles through overstretching, which is why it is also known as an overstretched honeycomb. Compared to hexagonal honeycombs, overstretched honeycombs do not take on a saddle shape when bent, meaning they possess excellent bending deformation capabilities.

### 2.2. Three-Point Bending Test on Honeycomb

**Figure 2:**
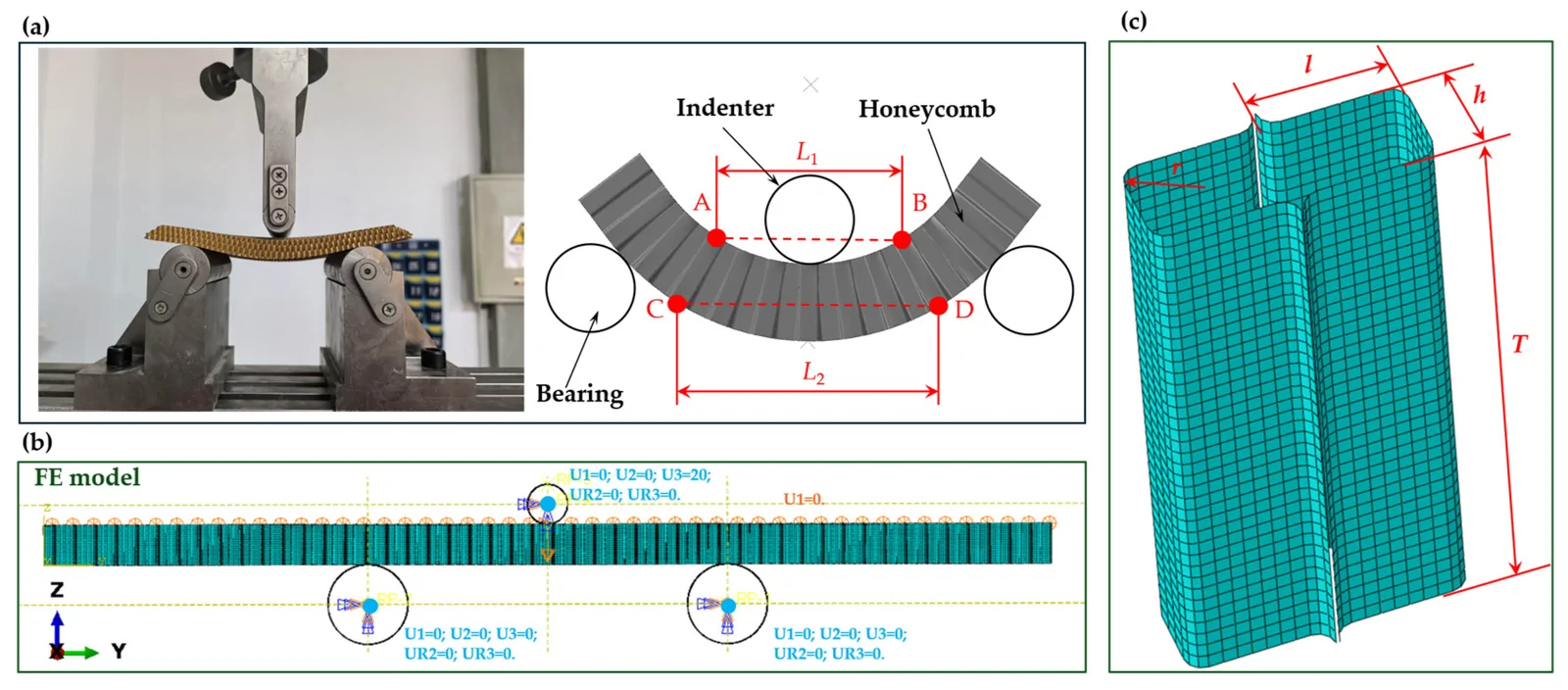
Three-point bending test; (**a**) Object model; (**b**) FE model; (**c**) Single-cell honeycomb FE model.

The three-point bending test on the honeycomb was conducted using a universal testing machine. In this experiment, two bearings with a diameter of 20 mm and an indenter with a diameter of 10 mm were used. Both the bearings and the indenter can rotate around a fixed axis. Before experiment, mark four points—A, B, C, and D—on the honeycomb, as shown in Figure 2. Before loading, ensure that AB and CD are of equal length and parallel to the end face of the honeycomb. During the loading process, the loading rate was set to 1 mm per minute, and the DIC was used to collect real-time data on the lengths of the secant lines AB and CD. Let the lengths of AB and CD be denoted by *L*_1_ and *L*_2_. Within the small area of the honeycomb, changes in *L*_1_ and *L*_2_ can indicate the extent to which the honeycomb’s end face is compressed or stretched. By comparing the changes in *L*_1_ and *L*_2_ obtained from experimental data and simulations, we can assess the accuracy of the finite element model in calculating the deformation of the honeycomb.

### 2.3. Finite Element Model of a Honeycomb

**Figure 3:**
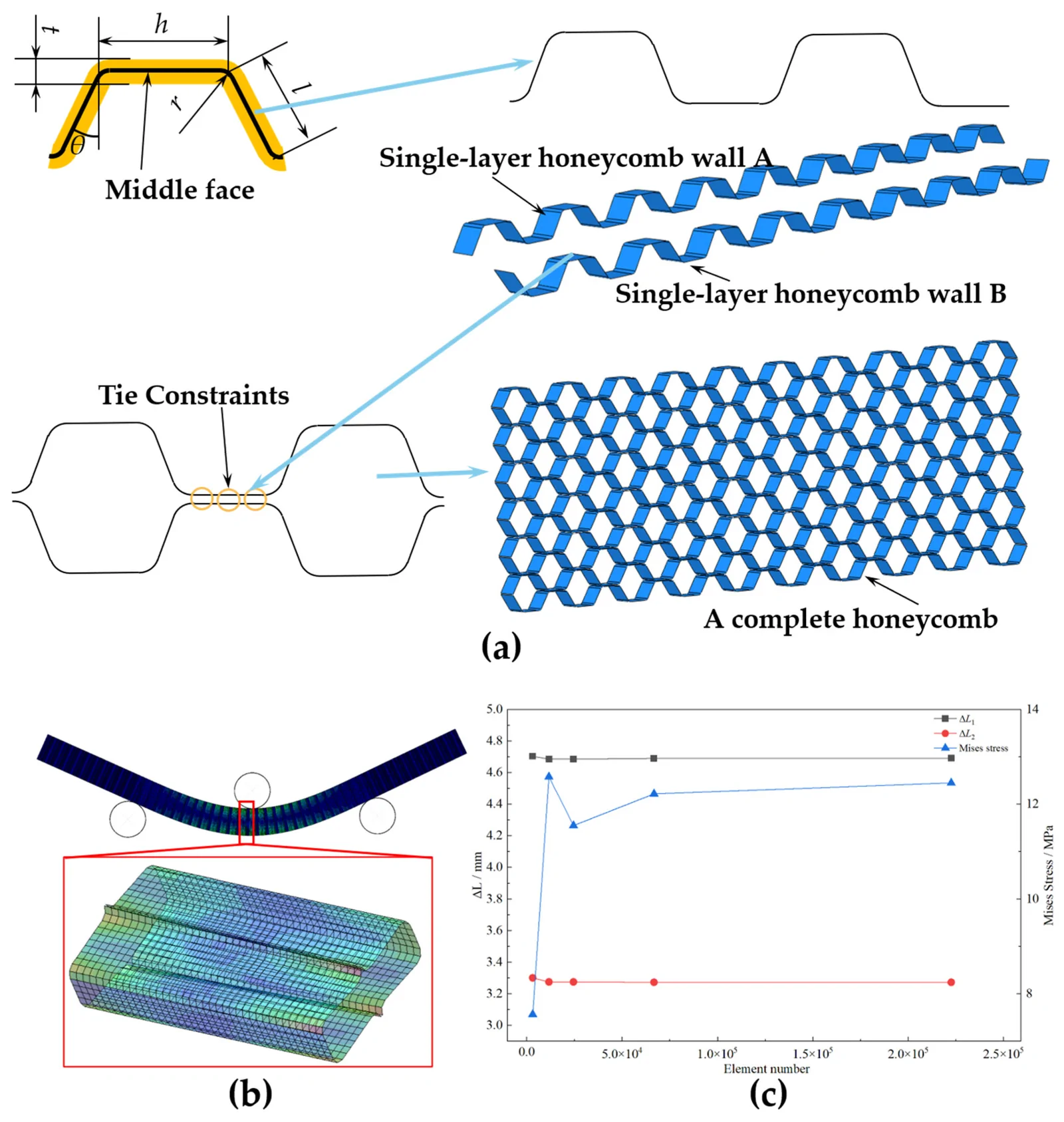
Honeycomb finite element model; (**a**) modeling method of FE model; (**b**) mesh generation of FE model; (**c**) results of grid sensitivity analysis.

The finite element model of the honeycomb core was established using ABAQUS 2025, as shown in Figure 2b,c and Figure 3a. The nominal thickness of aramid paper typically ranges from 0.05 to 0.75 mm, and the nominal length of the honeycomb cell walls ranges from 3.2 to 9.5 mm, making it a typical thin-walled structure. Therefore, a shell model was used to create a scaled model of the honeycomb core, which was then meshed using S4R elements. And a detailed mesh convergence study with 5 sets of element sizes from 0.4 mm to 6.4 mm was supplemented, with the number of elements varying from 200,000 to 3000. By comparing the Δ*L* and Mises stress, we finally selected the element size of 0.4 mm, as shown in Figure 3b,c. For ease of analysis, the modeling was based on the mid-plane of the honeycomb cell walls, with geometric parameters as shown in Table 2. Based on the manufacturing process of aramid paper honeycombs, single-layer honeycomb walls A and B were created separately. they are mirror-symmetric to each other. During the bending process of the honeycomb core, the delamination of the aramid paper pores was not considered. Therefore, a tie constraint was applied at the double-wall thickness to represent the adhesion between single-layer honeycomb walls A and B. Since the support can rotate around a fixed axis in the experiment, the friction between the mold and the honeycomb can be ignored in the contact settings of the simulation, and only the setting that the honeycomb and the mold cannot penetrate each other in the normal direction is retained. During the manufacturing process, the bending of the honeycomb core can be regarded as a quasi-static process. Therefore, a static analysis step is used for simulation.

## 3. Results and Discussion

### 3.1. Geometric and Mechanical Response of Honeycomb Under Bending Conditions

**Figure 4:**
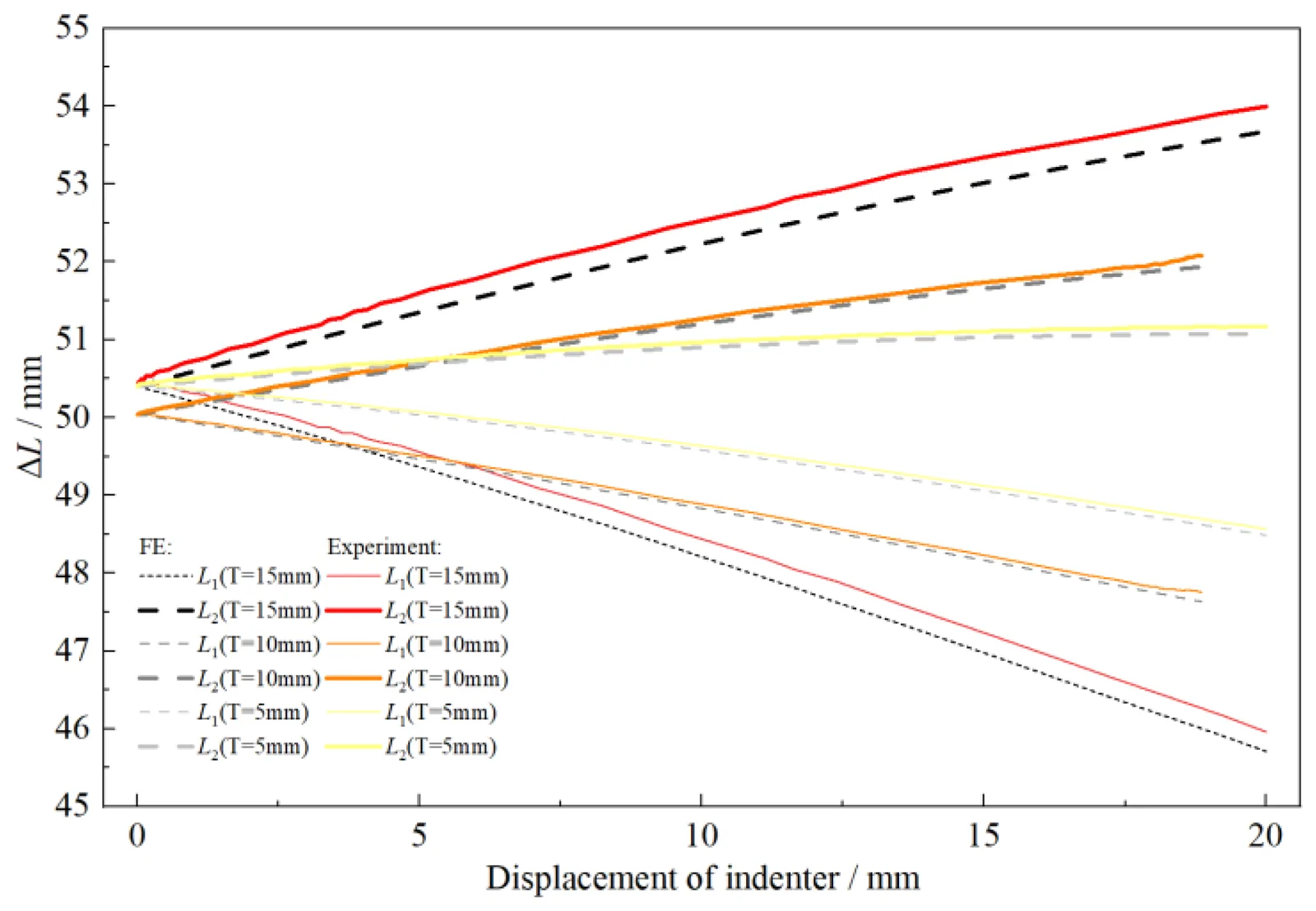
Comparison of S-L curves from DIC testing and simulation.

Figure 4 shows how *L*_1_ and *L*_2_ change as the pressure head decreases. As can be seen from the L-S curve, the compression and tensile strains at the end faces of a honeycomb are not the same when the honeycomb is bent. This is due to the fact that the compressive and tensile moduli of the honeycomb differ in the two directions. At the same time, this implies that as the honeycomb cell structure changes, the trends in its macroscopic mechanical properties also vary.

The simulated values for *L*_1_ and *L*_2_ do not exactly match the experimental values. Analysis shows that the discrepancy between the simulation results of the three-point bending test on the honeycomb and the experimental results stems, first, from the fact that the friction properties between the honeycomb and the fixture in the finite element model are not accurate. Second, due to the influence of the manufacturing process, the material of the honeycomb and the geometry of the individual cells are not entirely consistent. Both of these sources of error are unavoidable. The maximum error between the simulated and experimental values for both *L*_1_ and *L*_2_ is less than 10%, demonstrating that the finite element model accurately and reliably simulates the bending deformation of the honeycomb.

### 3.2. Investigation on Cell Angle Variation and Prediction Model of Honeycomb Under Bending

**Figure 5:**
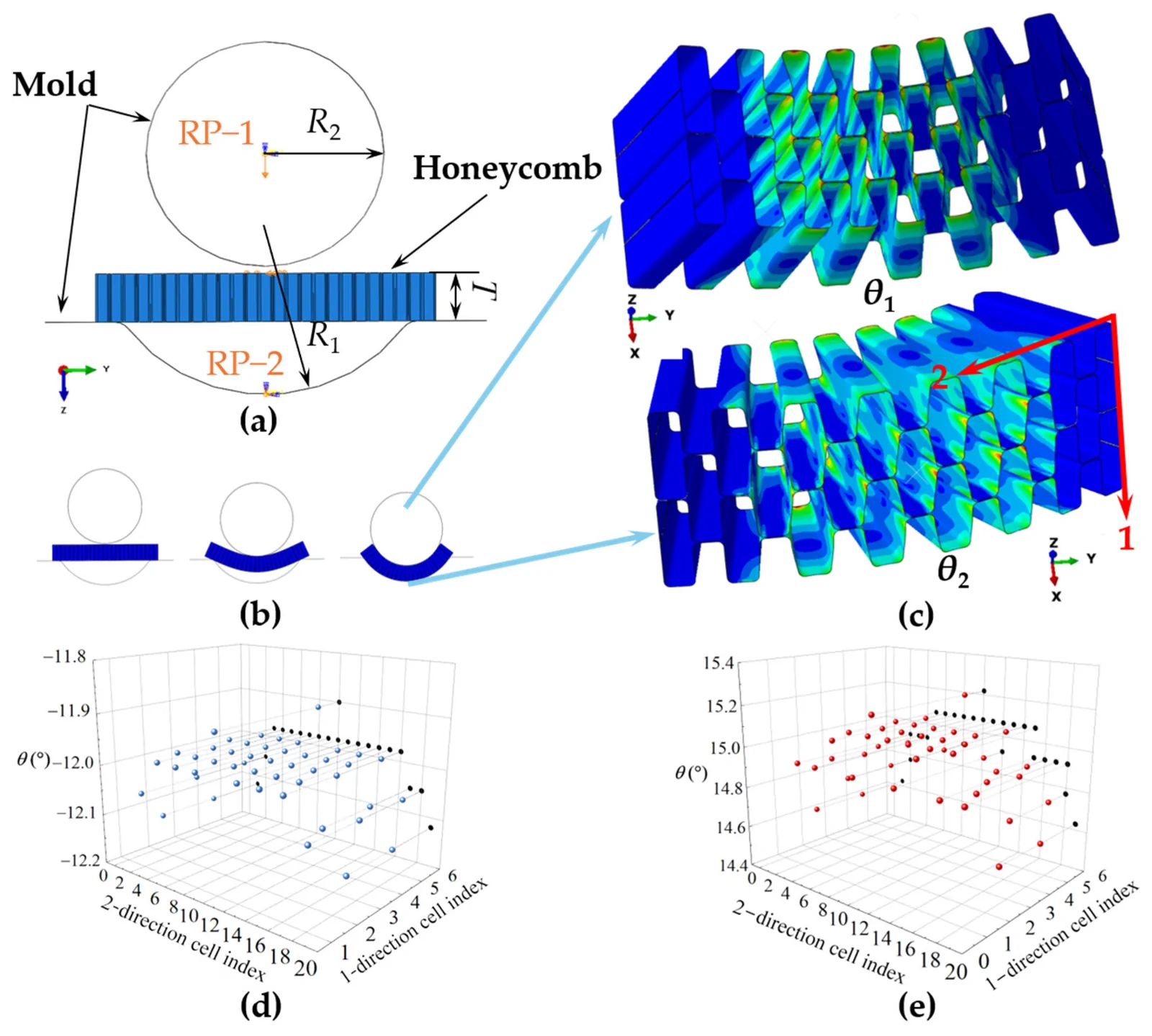
In-plane distribution law of honeycomb cell deformation; (**a**) finite element model of honeycomb bending; (**b**) honeycomb bending process; (**c**) definitions of *θ*_1_ and *θ*_2_; (**d**) in-plane distribution of *θ*_1_; (**e**) in-plane distribution of *θ*_2_.

It is difficult to determine the curvature of a honeycomb at any given moment during loading using a three-point bend. Therefore, this chapter will design a finite element model that uses a mold to bend the honeycomb into a specified curvature, as shown in Figure 5a. The finite element model consists of a punch, a die, and the honeycomb, all of which are represented as shell models. The thickness of the honeycomb is *T*. The punch is a circle with radius *R*_2_, the die is an arc with radius *R*_1_, and *T* = *R*_1_ − *R*_2_. The surface roughness of the tempered glass mold used in the experiment is in the range of Ra 0.02–0.05 μm, which makes it difficult to provide frictional resistance. In addition, during the honeycomb bending process, almost 90% of the part is in a non-contact state, and it only turns into full contact after the honeycomb is completely bent into place. Therefore, the contact between the honeycomb and the die is assumed to be frictionless. When unloaded, the honeycomb is straight. After loading is applied, the honeycomb bends and conforms completely to the molds. For the sake of simplicity, define the angle of the cell adjacent to the punch cavity as *θ*_1_, and the angle of the cell adjacent to the die face as *θ*_2_, as shown in Figure 5c.

The angle values of the cells in the curved regions at both ends of the honeycomb were extracted from the simulation results, as shown in Figure 5d,e. When the honeycomb bends, the angles of the cells at different positions on a cross-section of the same thickness are essentially consistent. This implies that when the curvature of the honeycomb remains constant, the cells within the curved region undergo identical deformation. Therefore, it can be concluded that on a cross-section of the same thickness, the change in cell angle is independent of the cell’s position. Based on the above pattern, in subsequent analysis and discussion, a single cell can be selected as a representative for analysis.

**Figure 6:**
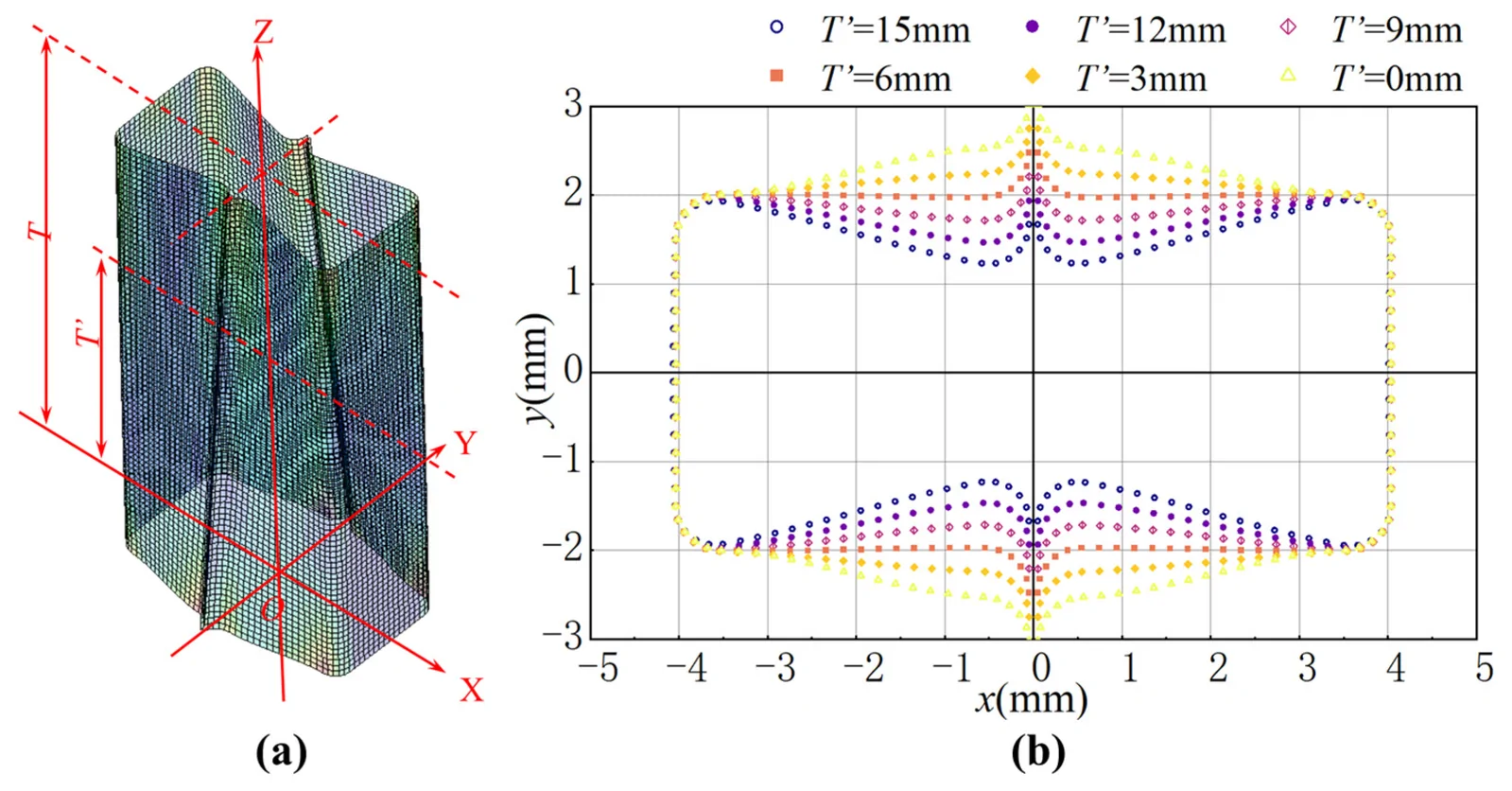
Deformation law along the cross-section of the cell; (**a**) establish a Cartesian coordinate system with the cell center as the origin; (**b**) the nodes of the cell are projected onto the XY plane.

**Figure 7:**
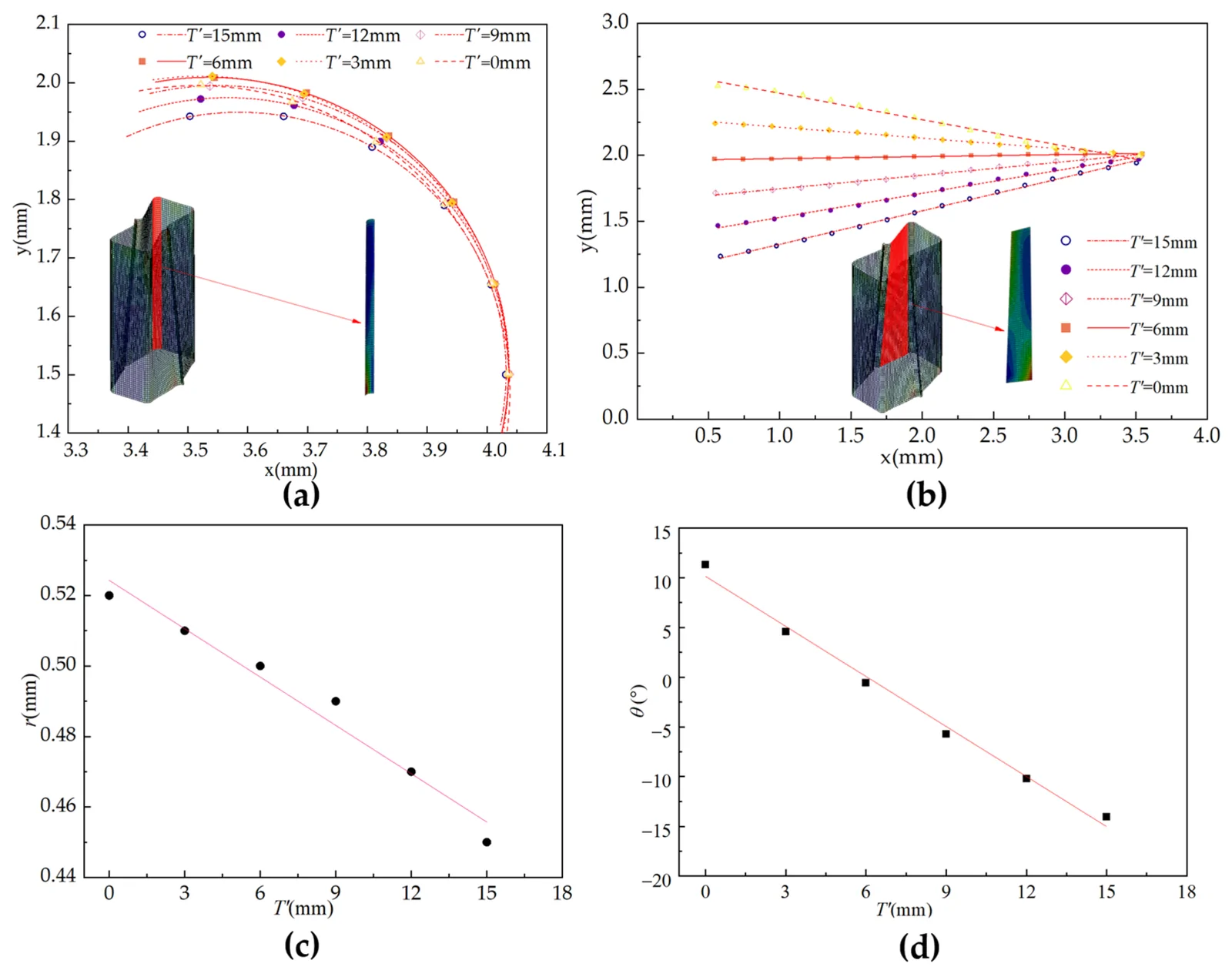
Analysis of cell deformation along the cross-section; (**a**) cell filet radii at cross-sections of different thicknesses; (**b**) cell walls in cross-sections of varying thicknesses; (**c**) pattern of changes in the radius of cell corner rounding with thickness across the cross-section; (**d**) pattern of changes in the angle of cell with thickness across the cross-section.

In Figure 6a, a Cartesian coordinate system is established with the center of the cell adjacent to the die as the origin, and the *Z*-axis parallel to the cell’s centerline. Samples are taken at 3 mm intervals in the direction of the cell thickness, and the nodes of the mesh model are projected onto the XY plane, as shown in Figure 6b. The results indicate that cell deformation is primarily concentrated at the single-layer walls, while it remains largely unchanged at the double-layer walls. To more accurately assess the deformation of the elements, data from the single-layer wall and the filet sections in Figure 6b are extracted separately in Figure 7 for further analysis.

Figure 7a shows the filet at different thicknesses within the same cell. The fitted curve indicates that, despite variations in the filet, it generally retains the shape of a circular arc. Figure 7c illustrates the variation in the filet radius *r* with the thickness position *T*′. The fitting results suggest that the variation in the filet radius follows a roughly linear trend. As *T*′ varies, the relative change in the filet radius is only 13%. Therefore, this paper will disregard the equivalent effect of filet changes on honeycomb homogenization. Figure 7b shows the deformation of the single-layer wall. The fitting results indicate that the single-layer wall remains essentially straight during cell deformation. This implies that the flexural deformation of the single-layer wall can be neglected when analyzing cell deformation. Additionally, the tangent of the slope of the fitted line in Figure 7b can be used to characterize the cell angle. The fitting results in Figure 7d show that the cell angle *θ* also varies linearly with *T*′. Therefore, given the cell angle at the end face of the honeycomb, we can use Equation (1) to calculate the cell angle at any position corresponding to a given value of *T*′.

(1) $$\theta =kT^{\prime }+\theta_{1}$$

**Figure 8:**
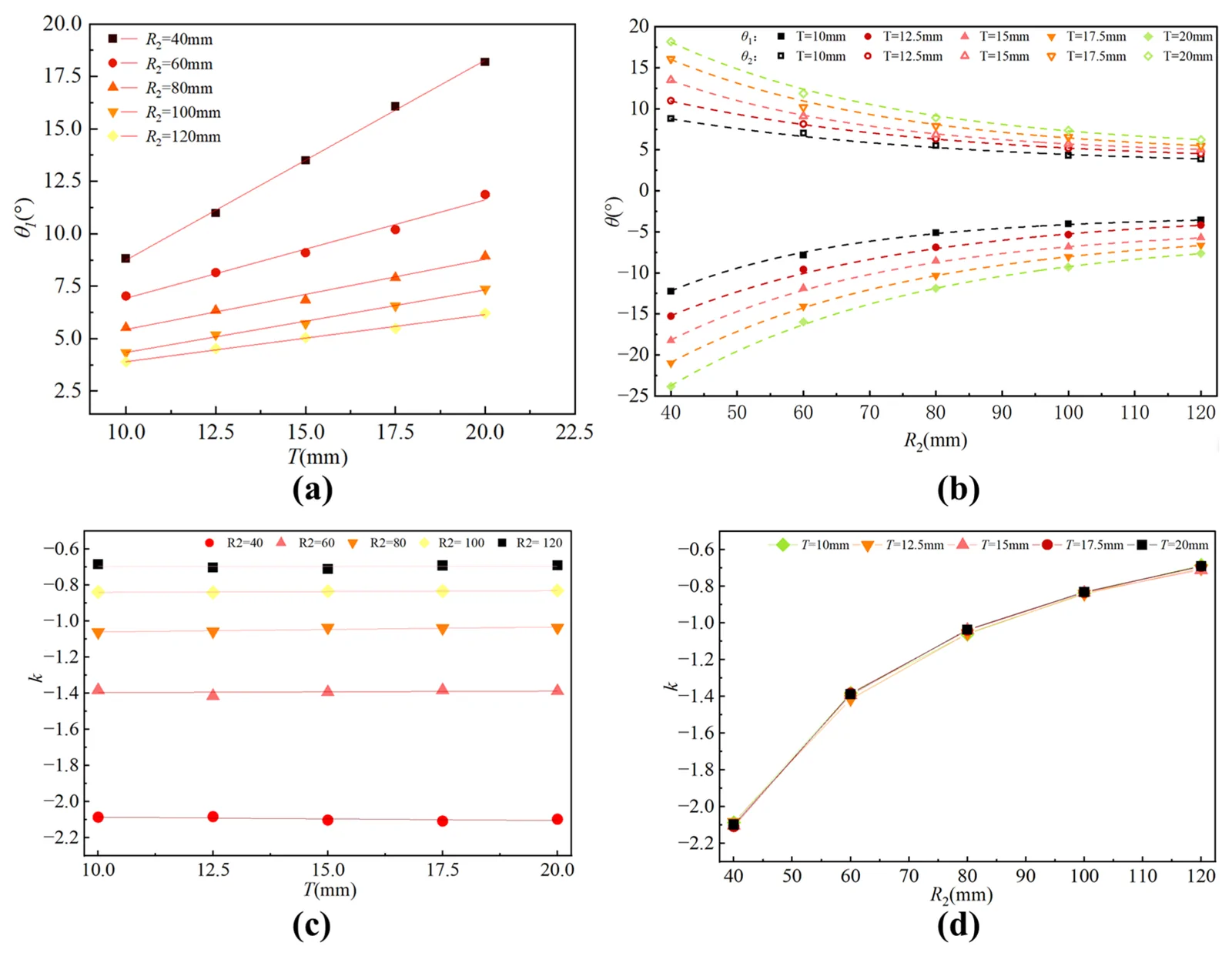
Cell deformation laws of honeycombs with different curvatures and thicknesses; (**a**) curve of *θ*_1_ versus honeycomb thickness *T* for different bending radii *R*_2_; (**b**) curves for *θ*_1_ versus bending radius *R*_2_ and *θ*_2_ versus bending radius *R*_2_ for different honeycomb thicknesses *T*; (**c**) curve of slope *k* versus honeycomb thickness *T* for different bending radii *R*_2_; (**d**) curve of slope *k* versus radius of curvature *R*_2_ for different cell thicknesses *T*.

For a given total thickness *T*, the angle between the cells on the end face of a honeycomb varies depending on the bending radius. Similarly, for a given bending radius, the angle between the cells on the end face of a honeycomb varies depending on the thickness. By designing a test matrix, we performed a simulation analysis to investigate the variation patterns of the angle of inclination and the slope *k* of the linear function for end-face cells of honeycomb cores under different initial thicknesses and curvatures. In Figure 8, the simulation data is fitted, and the minimum value of the Pearson correlation of the fitting curve is 0.93, which meets the requirements of fitting accuracy.

As can be seen from Figure 8, for all bending radii, the angle of the honeycomb end-face cells increases linearly with increasing initial thickness. For all initial thicknesses, the angle of the honeycomb end face exhibits a negative exponential relationship with the bending radius. If the angle of the honeycomb end face *θ*_1_ is expressed as a two-variable function of the initial thickness *T* and the bending radius *R*, then its partial derivative should satisfy Equation (2).

(2) $$\left\{\begin{array}{c} \frac{\partial \theta_{1}}{\partial T}=a_{1}e^{-R_{2}/b1}+a_{2}e^{-R_{2}/b2}+\ldots \ldots \\ \frac{\partial \theta_{1}}{\partial R}=a_{1}^{\prime }Te^{-R_{2}/b_{1}}+a_{2}^{\prime }Te^{-R_{2}/b_{2}}+\ldots \ldots \end{array}\right.$$

For all bending radii, the slope *k* is insensitive to changes in the initial thickness. However, for all initial thicknesses, the slope *k* behaves as a negative exponential function of the bending radius. Therefore, it can be concluded that the slope *k* is related solely to the bending radius.

In summary, we may express *θ*_1_ as in Equation (3) and *k* as in Equation (4).

(3) $$\theta_{1}=a_{1}Te^{-R_{2}/b_{1}}+a_{2}Te^{-R_{2}/b_{2}}+\ldots \ldots$$

(4) $$k=c_{1}e^{-R_{2}/d_{1}}+c_{2}e^{-R_{2}/d_{2}}+\ldots \ldots$$

Substituting Equations (3) and (4) into Equation (1) yields Equation (5). When the honeycomb structure is subjected to bending, Equation (5) expresses the cell angle as a function of the bending radius, the total thickness of the honeycomb, and the thickness-direction cross-sectional value.

(5) $$\theta =\left(c_{1}e^{-R_{2}/d_{1}}+c_{2}e^{-R_{2}/d_{2}}+\ldots \ldots \right)\cdot T^{\prime }+a_{1}Te^{-R_{2}/b_{1}}+a_{2}Te^{-R_{2}/b_{2}}+\ldots \ldots$$

The simulation dataset obtained from the test matrix was subjected to nonlinear polynomial fitting according to Equation (5) to determine the constant term in Equation (5), thereby simplifying it to Equation (6).

(6) $$\theta =\left(1.96e^{-R_{2}/27.2}+0.55e^{-R_{2}/198.28}\right)T+(-5.22e^{-R_{2}/24.49}-1.37e^{-R_{2}/164.59})T^{\prime }$$

Similarly, if *R*_1_ is taken as one of the variables, the cell angle can be predicted using Equation (7).

(7) $$\theta =\left(-1.51e^{-R_{1}/15.47}-1.13e^{-R_{1}/91.71}\right)T+(2.83e^{-R_{1}/24.49}+1.25e^{-R_{1}/164.59})T^{\prime }$$

**Figure 9:**
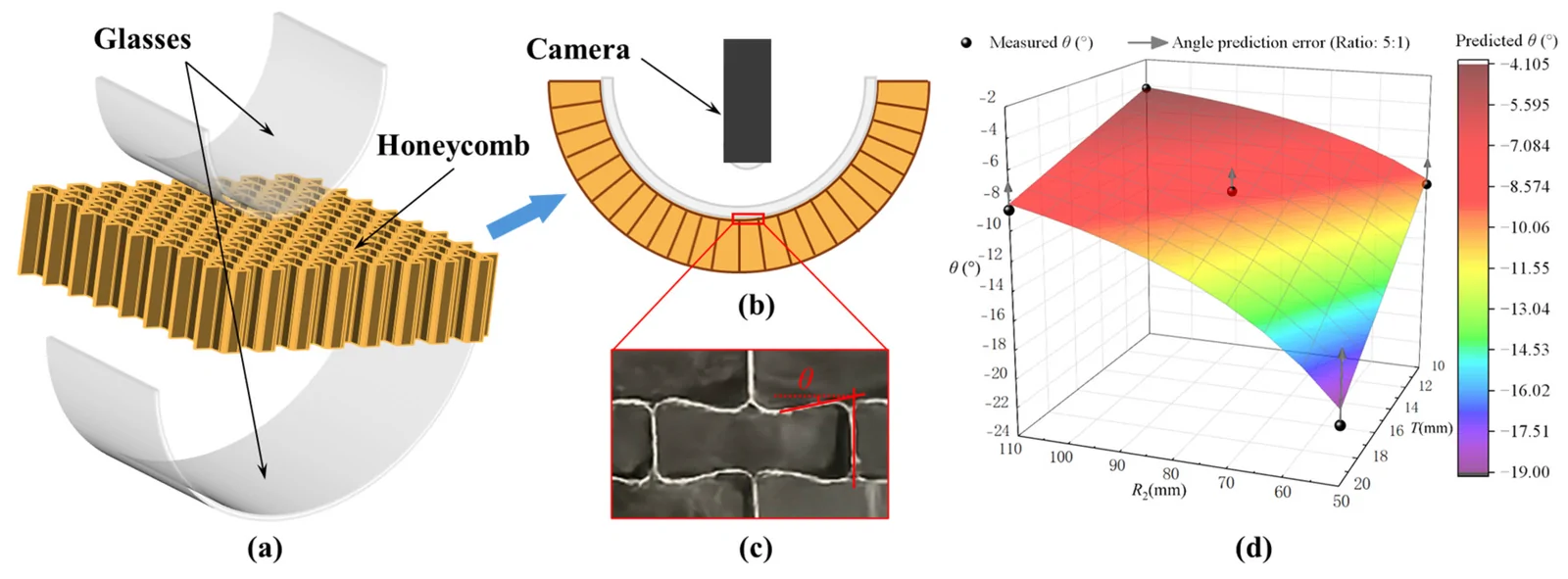
Comparison between experimental results and predicted results of cell deformation; (**a**) bending honeycomb using a glass mold; (**b**) capturing images of cells; (**c**) calibrating the *θ* angle; (**d**) a comparison of several sets of discrete measured data with the predictions from the semi-empirical model, along with an error analysis.

Using Equations (6) and (7), given the honeycomb’s radius of curvature and initial thickness, we can predict the shape of the cells at various locations throughout the honeycomb. Therefore, these two equations constitute semi-empirical models for predicting cell deformation. Furthermore, experiments have demonstrated the accuracy of the semi-empirical model’s predictions, as shown in Figure 9.

A punch and a die were fabricated from transparent glass to bend the honeycomb to a specified curvature, as shown in Figure 9a. Because the glass is transparent, a camera can clearly capture the outline of the honeycomb cells in close contact with the die surface, as shown in Figure 9b. Since zooming in or out on the image does not alter the angle values, the change in cell angle can be measured by examining the enlarged cell outline, as shown in Figure 9c. Figure 9d presents a comparison of several sets of discrete measured data with the predictions from the semi-empirical model, along with an error analysis. Larger errors occur when the honeycomb is thicker and the bending radius is smaller. The maximum relative error in the predicted angle is 5.05%, which is within an acceptable range. These results demonstrate that the semi-empirical model accurately predicts changes in the angles of bent honeycomb cells.

## 4. Conclusions

In this work, to solve the modeling problem of honeycombs in curved honeycomb sandwich structures, we conducted a study on the equivalent method for curved honeycombs based on the theory that the homogenized equivalent properties of honeycombs are functions of their cell structures. Through finite element simulations, we found that when the honeycomb is bent, the deformation response of its cells can be summarized as a function of the bending radius and the initial thickness of the honeycomb. Combining a large amount of simulation data, we gave the specific expression for the deformation response of honeycomb cells of a specific type. Using this formula, the cell shape at various locations under a specific bending configuration of the honeycomb can be quickly predicted. The results of this study provide a theoretical basis for the fine homogenized equivalent modeling of curved honeycombs.

Although this study has gained meaningful insights, some limitations still need to be addressed in future work. When considering the influence on the cell deformation, the above semi-empirical model only takes into account three parameters—the bending radius and the honeycomb thickness. Further research is required to explore the influence of other honeycomb parameters on the cell deformation, with the aim of applying these research results to the prediction of the honeycomb cell deformation of different specifications and materials. Meanwhile, further research is needed to demonstrate whether the inconsistent deformation of some honeycomb cells caused by manufacturing defects will have an impact on the overall performance of the honeycomb homogenization equivalent.

## Figures and Tables

**Table 1:** Mechanical properties of honeycomb matrix materials.

Material	Young’s Modulus (MPa)	Shear Modulus (MPa)	Poisson’s Ratio
Resin-cured aramid paper	2821.78	1077.02	0.31

**Table 2:** Specifications and parameters of aramid paper honeycomb.

Material	*t* (mm)	*l* (mm)	*h* (mm)	*T* (mm)	*r* (mm)	*θ* (°)
Aramid paper honeycomb	0.1	3.75	2.6	15	0.5	0

## Data Availability

The raw data supporting the conclusions of this article will be made available by the authors on request.

## References

[1] Masters I.G., Evans K.E. (1996). Models for the Elastic Deformation of Honeycombs. Compos. Struct..

[2] Bezazi A., Scarpa F., Remillat C. (2005). A Novel Centresymmetric Honeycomb Composite Structure. Compos. Struct..

[3] Balawi S., Abot J.L. (2008). A Refined Model for the Effective In-Plane Elastic Moduli of Hexagonal Honeycombs. Compos. Struct..

[4] Ajdari A., Nayeb-Hashemi H., Canavan P., Warner G. (2008). Effect of Defects on Elastic–Plastic Behavior of Cellular Materials. Mater. Sci. Eng. A.

[5] Mukhopadhyay T., Adhikari S. (2016). Equivalent In-Plane Elastic Properties of Irregular Honeycombs: An Analytical Approach. Int. J. Solids Struct..

[6] Bargmann S., Klusemann B., Markmann J., Schnabel J.E., Schneider K., Soyarslan C., Wilmers J. (2018). Generation of 3D Representative Volume Elements for Heterogeneous Materials: A Review. Prog. Mater. Sci..

[7] Qiu C., Guan Z., Jiang S., Li Z. (2017). A Method of Determining Effective Elastic Properties of Honeycomb Cores Based on Equal Strain Energy. Chin. J. Aeronaut..

[8] Al-Masri A., Khanafer K. (2026). Multi-Scale Thermal Homogenization of PCM-Filled Aluminum Honeycomb Structures for Electronic Device Thermal Management. Int. J. Heat Mass Transf..

[9] Carrera E., Salem K.A., Augello R. (2025). The Component-Wise to Evaluate Homogenized Methods in Layer-Wise Approaches for Honeycomb Sandwich Panels. Compos. Struct..

[10] Ma P., Zhang J., Quan T. (2024). Prediction of In-Plane Mechanical Properties of Auxetic Honeycombs Based On Machine Learning. Acta Mater. Compos. Sin..

[11] Yang R., Li S., Sun S., Niu B., Wang R., Han X.c. (2024). Nonlinear Performance Analysis and Rapid Prediction of Out-of-Plane Deformation in Graded Honeycombs. Thin-Walled Struct..

[12] Yan J.-H., Li F.-L. (2026). Vibro-Acoustic Prediction and Optimization of Fiberglass NPR Honeycomb Sandwich Structure in Thermal Environment Based on Machine Learning. Thin-Walled Struct..

[13] Zhao L., Rajput A., Sharma N., Shen X., Kumar P. (2025). Bending Performance of Aluminium Honeycomb Sandwich Composites vs. GRP: A Comparative and Parametric Investigation. Results Eng..

[14] Ren J.W., Zhou Y.L., Gao W.B. (2023). Bending response and failure characteristics of nomex honeycomb sandwich with continuous composite face sheet encasement. J. Sandw. Struct. Mater..

[15] Li S., Yang R., Sun S., Niu B., Liu R. (2025). Freeform Surface Morphing Using Honeycomb Plates: Gaussian Curvature Control. Int. J. Mech. Sci..

[16] Nguyen-Van V., Ha N.S., Nguyen-Xuan H., Lin X., Xie Y.M., Lu G. (2025). Effect of Honeycomb Core Cell Geometry on the Sandwich Tube for Internal Blast Loading. Eng. Struct..

[17] Harkati A., Boutagouga D., Harkati E., Bezazi A., Scarpa F., Ouisse M. (2020). In-Plane Elastic Constants of a New Curved Cell Walls Honeycomb Concept. Thin-Walled Struct..

[18] Harkati E., Daoudi N., Bezazi A., Haddad A., Scarpa F. (2017). In-Plane Elasticity of a Multi Re-Entrant Auxetic Honeycomb. Compos. Struct..

[19] Harkati A., Harkati E.H., Bezazi A., Scarpa F., Ouisse M. (2021). Out-of-Plane Elastic Constants of Curved Cell Walls Honeycombs. Compos. Struct..

[20] Bezazi A., Remillat C., Innocenti P., Scarpa F. (2008). In-Plane Mechanical and Thermal Conductivity Properties of a Rectangular–Hexagonal Honeycomb Structure. Compos. Struct..

